# Generative artificial intelligence: a historical perspective

**DOI:** 10.1093/nsr/nwaf050

**Published:** 2025-02-21

**Authors:** Ran He, Jie Cao, Tieniu Tan

**Affiliations:** New Laboratory of Pattern Recognition, Institute of Automation, Chinese Academy of Sciences, Beijing 100190, China; School of Intelligence Science and Technology, Nanjing University, Nanjing 210008, China; New Laboratory of Pattern Recognition, Institute of Automation, Chinese Academy of Sciences, Beijing 100190, China; New Laboratory of Pattern Recognition, Institute of Automation, Chinese Academy of Sciences, Beijing 100190, China; School of Intelligence Science and Technology, Nanjing University, Nanjing 210008, China

**Keywords:** artificial intelligence, foundation model, generative method

## Abstract

Generative artificial intelligence (GAI) has recently achieved significant success, enabling anyone to create texts, images, videos and even computer codes while providing insights that might not be possible with traditional tools. To stimulate future research, this work provides a brief summary of the ongoing and historical developments in GAI over the past 70 years. The achievements are grouped into four categories: (i) rule-based generative systems that follow specialized rules and instructions, (ii) model-based generative algorithms that produce new content based on statistical or graphical models, (iii) deep generative methodologies that utilize deep neural networks to learn how to generate new content from data and (iv) foundation models that are trained on extensive datasets and capable of performing a variety of generative tasks. This paper also reviews successful generative applications and identifies open challenges posed by remaining issues. In addition, this paper describes potential research directions aimed at better utilizing, understanding and harnessing GAI technologies.

## Teaser

This paper reviews the historical milestones, successful applications, and remaining challenges in generative artificial intelligence over the past seven decades.

## INTRODUCTION

Generative artificial intelligence (GAI) refers to a group of AI algorithms and models that are capable of producing new content, including texts, images, videos and problem-solving strategies, with human-like creativity and adaptability. The past few years have witnessed unprecedented advancements in GAI. Notably, the AI system ChatGPT [1] can communicate with humans in over 80 languages, and it can be used to perform almost any task for which text responses are appropriate. The capabilities of ChatGPT facilitate its use for generating visual, audio and even multimodal content. This success stems from the development of GAI over half a century. For instance, representative events include the rise of deep learning, transformer architectures and foundation models.

This work presents a systematic review of GAI from a historical perspective. The scope of the work includes modern GAI, which is realized through programmable computers. We review the history from the origin to the present, highlighting milestone events and organizing them into four stages.

1. *Rule-based generative systems*. Computerized methods for autonomous generation emerged in the 1950s, followed by computer programs that are capable of generating data. These programs typically generate data by following the rules designed by human experts. During this period, expert systems achieved early success in some specific tasks.
2. *Model-based generative algorithms*. Researchers designed generative algorithms based on statistical or physical models. Hence, GAI came to include studies in machine learning, neural networks, computer graphics, computer vision, etc. Then, various generative applications built upon these studies were introduced. Among these examples, technologies such as computer animation generation became reliable for practical use, and have started to replace human efforts in content generation.
3. *Deep generative methodologies*. Benefiting from the growth in computational power and data resources, deep neural networks [2,3] have demonstrated superior power in content generation [4]. Then, deep generative models, including autoregressive-based [5] and diffusion-based [6] models, have been introduced and served as the basis for numerous practical applications until the present. Moreover, researchers of computer graphics developed deep learning–based approaches [7] that show improved capability and scalability in open environments.
4. *Foundation models*. The advent of generative pretrained transformers (GPTs) [8–10], which are a prominent family of foundation models, represents a significant revolution in GAI. Such models leverage deep learning techniques, but they are characterized by their large scale in terms of model size and training data. The strategy of scaling up yields unprecedented advantages, including high-quality content generation, natural interactions and versatility across tasks. Consequently, foundation models have become the driving force of content generation across various applications.

The rise of GAI has revolutionized the production of content and services to create multimedia data and other content types, such as plans, codes and proteins. The number of industries adopting GAI technologies has been increasing rapidly, especially since foundation models became popular. Today, traditional sectors such as manufacturing, developing industries such as autonomous driving [11] and emerging fields such as molecular design [12] have seen successful implementations based on generative approaches.

**Figure 1.**
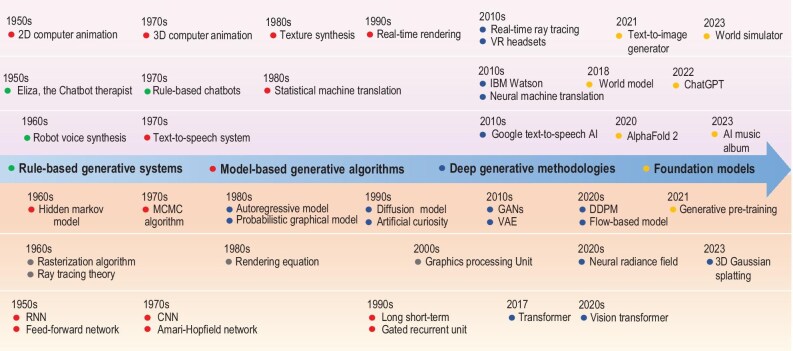
Timeline of the development of GAI methods and applications.

A representative timeline is shown in Fig. 1, tracing the development trajectory of GAI methods and applications. In the remaining parts of this paper, we detail representative approaches, discuss the strengths and limitations of different kinds of generative technologies, and introduce successful generative applications in various fields. In addition, we summarize the open challenges and possible future directions.

## RULE-BASED GENERATIVE SYSTEMS

**Figure 2.**
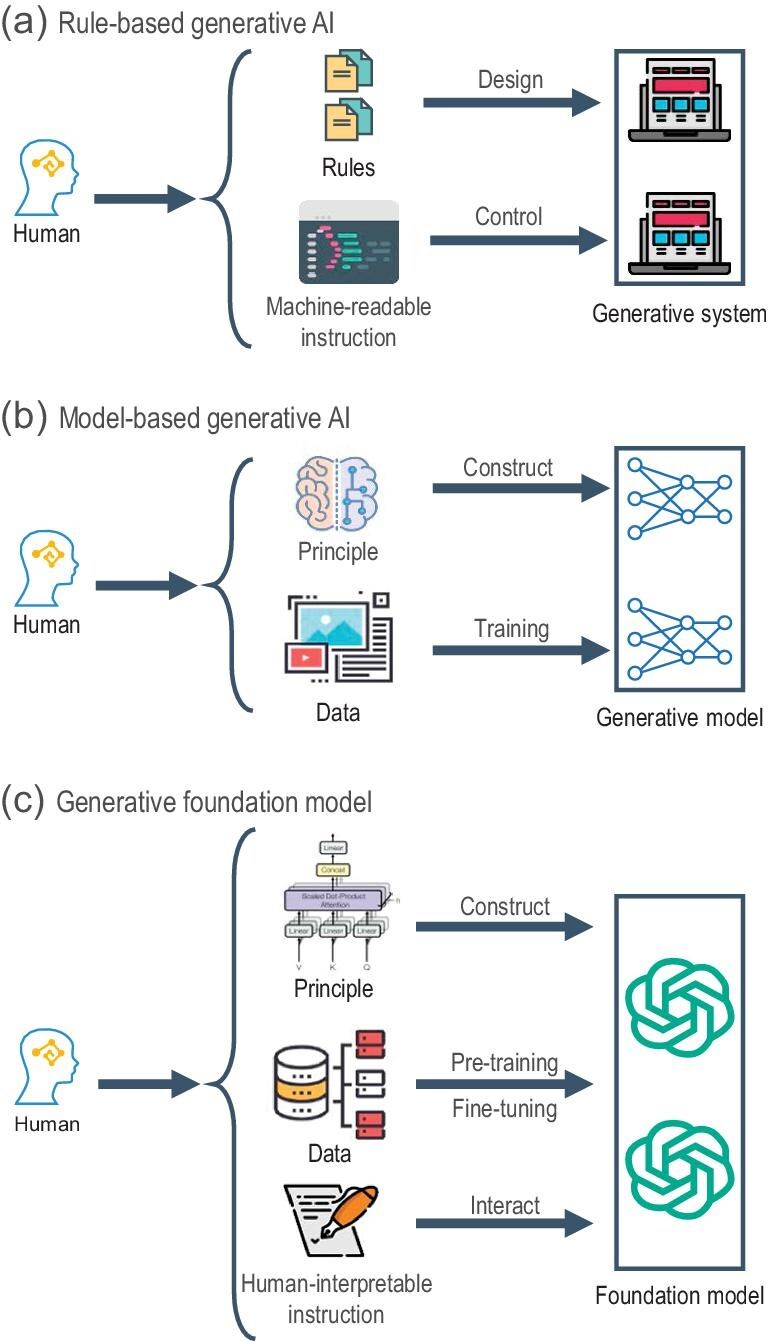
Evolution of design principles in GAI.

Studies of automatic data generation can be traced back to the 1950s, when symbolic AI emerged. During that period, as shown in Fig. 2a, researchers designed rules based on their expertise and implemented programs to execute generative tasks according to such rules. Generally, such a program [13] consisted of two primary components, namely, a generation engine and an interpreter.

The function of the generation engine is to generate data through various formulaic operations. It is structured around a knowledge base that includes rules and facts. Human experts design different types of rules and formulate them with distinct antecedent and consequent components [14]. Then, they embed the rules into the generation engine through coding in the form of symbolic descriptions. Notably, these rules are effective for one specific task. Therefore, subsequent research [15] has developed rules for more tasks, such as dialogue, translation, etc. On the other hand, the facts in the generation engine provide factual information that conveys assertions about propositions [13], properties [14], relations [16], etc. When an input signal that contains factual information is received, the generation engine traverses all the rules. Then, after the engine has identified a rule that matches the current fact, it takes actions following the rules and generates new facts. This process runs in a loop until the rules for concluding the generation process are satisfied.

The interpreter in the generative programs ensures that humans can understand the reasons behind the operations made by the generation engine. To achieve this goal, the interpreter translates the rules and all possible actions into explanations in a human-readable language [17]. The translation involves mapping the logical structure of the rules to descriptions that can be read by humans, ensuring that users can easily understand the reasoning behind the system’s decisions. Notably, the interpreter is also rule based, but these special rules are not directly involved in the generation process. Hence, the rule-based generative program is a ‘white box’. The interpreter can always provide straightforward explanations for the generated results even if the data generation process is complex. In addition, debugging and modification of generative programs are also interpretable.

Expert systems, designed based on user-defined rules, were widely applied in generative tasks that required specialized knowledge from the 1950s to the 1990s. Successful applications included but were not limited to chatbots, machine translation systems and speech synthesis systems. We examine each of these representative applications as follows.

One of the first AI chatbots, ELIZA, was introduced in 1966. It acts as a psychotherapist that responds to patients. ELIZA processes the text inputs via pattern matching and then seeks predesigned responses based on the rules. The success of this pioneering chatbot is rooted in the limited scope of discussion topics, where rules are quite effective for simulating human conversations. Subsequent studies [18] designed chatbots for more roles such as a schizophrenia patient. However, these rule-based chatbots had a limited ability to understand context and were only applicable to a specific task.

A machine translation system was first proposed in the late 1950s. It contained detailed linguistic and grammatical rules, as well as a fact base composed of linguistic knowledge. Subsequently, translation systems supported by rules from other computational experts and computational linguists were proposed. For instance, SYSTRAN [19], which was developed in 1968, served as a translation tool for web browsers until 2007.

The introduction of speech synthesis systems can be attributed to the system proposed by Fant *et al.* [20] in the 1960s. This system involved using linguistic and phonetic rules to model the characteristics of speech and connect speech segments. The synthesized speech fragments have a noticeable mechanical tone and are not fluent, differing significantly from natural human speech. Nevertheless, they are clear and easy to understand, thus meeting the requirements for certain practical applications.

Although rule-based generative programs have achieved notable applications, they always face an inherent challenge: scenarios outside predefined rules. For real-world applications, manually designed rules cannot consider all possible situations; thus, generative programs inevitably encounter situations beyond their capabilities. Moreover, in highly complex scenarios, the number of generative rules increases substantially, making the design and update processes prohibitively expensive.

## MODEL-BASED GENERATIVE ALGORITHMS

To overcome the abovementioned inherent issues of the rule-based approach, researchers have explored generative algorithms based on models grounded in certain principles. The concept behind this design, shown in Fig 2b, continues to be the de facto standard in GAI. Below we review the algorithms based on statistical machine learning and computer graphics due to their significant contributions to GAI.

### Statistical machine learning models

Statistical machine learning aims to design algorithms that can learn from data how to complete tasks, instead of following explicit rules. Generally, these approaches can be categorized into discriminative and generative models [21]. The former focus on learning to make predictions and decisions from data. In contrast, generative models aim to model the data distribution and then synthesize data through inference or sampling. Since the 1960s, several research approaches to data generation have emerged for generative modeling or approximate inference.

Generative modeling methods capture the characteristics of data distributions to construct statistical models explicitly or implicitly. In general, the most typical explicit approach entails probabilistic graphical models [22]. These models build graphs where the nodes are random variables that describe data, and the edges represent probabilistic relationships between variables. Building on this concept, generating data can be interpreted as the process of inferring the unknown part of nodes in the graph. The optimization process followed in applying these models uses likelihood maximization algorithms, most notably the expectation–maximization algorithm [23]. Moreover, these models typically postulate the Markov property [24], which states that the future state of data depends solely on its current state. Hidden Markov models [25] introduce latent variables when generating data, that is, the observable variables depend on the latent variables. Following this approach, Harris *et al.* [26] constructed probabilistic graphical models where each node is independent of non-neighbors given that its neighboring nodes are determined. This type of approach significantly reduces the complexity of modeling, making the data generation process computationally feasible. Subsequent studies [27] have incorporated latent nodes into the graph model and successfully generated sequential data. Li *et al.* [28] introduced Markov networks that enforce bidirectional and symmetric edges defined by potential functions. Such networks are widely utilized for generating high-dimensional data, such as images. Friedman *et al.* [29] developed Bayesian networks that use direct edges to represent dependencies between nodes, which is effective for generating new content based on partially known data.

Autoregressive models [5] constitute another type of explicit modeling approach, particularly suited for data consisting of sequential elements. The generation of elements follows a one-by-one approach, where the probability distribution of each element is estimated based on the previously generated values. Given the sequential nature of language and speech, autoregressive models appeared for their generation [30] in the 1980s. In the 1990s, autoregressive neural networks capable of processing and generating sequential data were subsequently proposed [31]. More recently, autoregressive generation approaches [8] have been extended to large-scale neural networks, paving the way for the emergence of foundation models such as GPTs [9].

Other studies have explored approaches to implicit data modeling. For example, normalizing flows [32] use a series of invertible transformations, i.e. flows, to convert a prior distribution into a complex data distribution. These methods do not explicitly estimate the data distribution, but instead present a probability density function for data generation. When stochastic transformations are applied, the generation process is considered to be the evolution of a diffusion model. The concept of the diffusion model was introduced by Jarzynski [6] in a study of non-equilibrium systems. Stein *et al.* [33] proposed a probabilistic approach to learn the diffusion process using a parameterized model. It has been demonstrated theoretically [34,35] that flow-based models and diffusion-based models can be amalgamated into a collection of differential equations [36]. Although the two types of approaches were not mainstream at that time, their successors, probabilistic diffusion models [37,38] and flow matching [39], gained significant prominence in the deep learning era.

In addition, another group of approaches leverages artificial neural networks for generative modeling. The term ‘artificial neural network’ originates from studies of nerve cells [40], while its mathematical inception is rooted in hierarchical linear regression [41,42]. In subsequent developments, artificial neural networks have advanced through the integration of non-linear transformations and the design of complex architectures, now known as deep learning. The basic computational unit in these networks is a neuron [43]; neurons connect to each other through nonlinear activation functions. Such nonlinearity allows these models to theoretically approximate any distribution given a sufficient number of neurons. Hence, many studies leverage artificial neural networks to model data distributions for generative tasks.

Many studies have explored the architectures of artificial neural networks to specify how the components of networks are organized and interact. Several classic network architectures have been proposed, and their design principles continue to influence modern deep neural networks. Feed-forward neural networks, introduced in the 1950s [44], were widely adopted due to their simplicity and effectiveness. Convolutional neural networks (CNNs) emerged in the 1970s [45]. Two-dimensional CNNs, as described by Zhang *et al.* [46], have become fundamental to image processing and generation with neural networks. Recurrent neural networks (RNNs) incorporate recurrent connections and internal memory, making them well suited for modeling and generating sequential data. The origin of RNNs can be traced back to the mathematical model in statistical mechanics introduced by Lenz [47] and Ising [48]. Kleene [49] conducted a formal analysis of RNNs and framed them within the context of neural networks. Subsequently, the Amari–Hopfield network [50,51] introduced the ability to learn and associatively recall data patterns. It provided a core architecture for storing and generating diverse types of data. Hochreiter and Schmidhuber [52] introduced long short-term memory to better manage the memory and forgetting mechanisms, leading to significant improvements in generating textual data.

Furthermore, several studies [53,54] extend the Amari–Hopfield network framework to explicitly model probability distributions. These models learn an energy function based on data, where lower values correspond to more probable data configurations. The restricted Boltzmann machine [55] was introduced in the 1980s. Researchers applied a two-layer neural network for hierarchical data modeling and used an energy function to determine the probability of neural states.

Backpropagation is the core technique used to train modern neural networks, including those designed for generative tasks. In the 1970s, Linnainmaa [56] developed a method to optimize the parameters of neural network-like models by recursively applying the chain rule to compute derivatives. Werbos [57] proposed applying this method to train artificial neural networks. Starting in the 1980s, this optimization method was commonly referred to by its current name, ‘backpropagation’ [58]. Through a series of studies [58,59], backpropagation has proven to be a general method enabling neural networks to learn useful representations.

The inference process of generative models produces data from trained models. In practice, direct inference is often impractical due to its mathematical intractability or excessive computational demands [60]. Consequently, existing methods generate data through approximate inference based on empirical priors and observed historical data. Such methods typically fall into two categories: stochastic approximation and variational inference.

In the realm of generative models, stochastic approximation aims to estimate the probability distribution of data gradually by random sampling. The most representative method is the Markov chain Monte Carlo algorithm [61,62], which constructs a Markov chain where each state corresponds to a data point embedded in the sample space. A carefully designed probability between the states ensures that the stationary distribution of a Markov chain approaches the data distribution. Brooks *et al.* [63] introduced efficient sampling strategies assuming that the data distributions are almost independent. Subsequent research has focused on improving the strategies of step size selection [64], sample selection [65] and efficient computing [66].

Variational inference was systematically introduced into machine learning in the 1990s [67,68]. The core concept is utilizing a tractable parameterized distribution, known as a variational distribution, to approximate the real-world distributions of data. The approximation error between the variational distribution and the true distribution can be effectively measured by an evidence lower bound [67]. Because of its flexibility, variational inference facilitates various generative tasks in complex scenarios.

When applied to generative tasks, the approaches based on statistical machine learning demonstrate better generalizability than rule-based generative systems. However, for these approaches, adapting to real-world situations remains challenging due to practical issues such as the curse of dimensionality. Nonetheless, some applications have achieved notable success. For instance, the rise of statistical machine translation, which replaced rule-based translation systems, occurred in the 1980s. This implies that the focus of GAI shifted from pursuing expert knowledge to collecting large-scale datasets. Later, in the 1990s, statistical approaches became mainstream for speech translation and synthesis until they were replaced by deep learning models. Moreover, statistical approaches have also been applied to visual content generation. But these applications are limited to specific tasks, such as texture synthesis and image fusion.

### Graphics-based models

Graphics-based methods focus on creating visual content through physical modeling. These approaches stem from Marr’s theory of vision [69], a paradigm for reconstructing the shape and appearance of real-world scenes. Within this framework, content generation is achieved through rendering, which refers to the process of combining materials, textures, lighting and other elements to produce visual effects. Recent studies [7,70] have incorporated deep generative learning into these methods, thus making them a part of GAI.

Graphics-based methods reconstruct three-dimensional (3D) scenes either explicitly or implicitly. Explicit representations, such as lines, point clouds [71] and voxels [72], are intuitive to humans and were widely adopted in the early stages of research. In contrast, implicit reconstruction methods use deep neural networks to encode scene information, enabling the rendering of images at arbitrary resolutions.

Rendering techniques can be broadly categorized into two types, namely, rasterization and ray tracing, both of which play crucial roles in GAI methods. These two rendering approaches offer complementary advantages. Rasterization algorithms [73–75] are highly efficient in utilizing hardware for fast rendering, whereas ray tracing algorithms [76–78] provide superior image quality at the cost of intensive computation.

There are also notable studies [79,80] of computing equipment customized for graphics-based methods. The first graphics processing unit (GPU) was presented in 1999, providing an accelerated framework for rasterization rendering. In 2004, Oh and Jung [81] proposed a GPU-based implementation of artificial neural networks. After two decades of development, GPUs have become foundational hardware devices for GAI. Moreover, researchers have focused on developing hardware-agnostic programming interfaces, such as OpenGL [79] and Direct3D [80]. These studies have standardized the pipeline of rasterization rendering used presently.

Graphics-based generative methods have led to widespread applications in computer animation. The first computer-animated film was released in 1958, marking the beginning of a gradual shift in film production, with generative methods gradually replacing manual techniques. In the mid-1990s, *Toy Story*, the first feature film created entirely with computer graphics, achieved significant commercial success. Since then, graphics-based generative methods have continued to evolve, becoming the core technology in video games, and enabling the production of highly realistic graphics and complex visual effects.

## DEEP GENERATIVE METHODOLOGIES

Notable achievements of GAI stem from the renaissance of deep learning [82,83]. In 2011, researchers [2] showed that increasing the depth of neural networks significantly improved their capacity to learn data representations, achieving superhuman performance in classification tasks. In the following year, subsequent studies [3,84] further corroborated the effectiveness of deep neural networks. Deep neural networks have since been widely applied to generative tasks [4,85]. These networks demonstrate powerful capabilities for understanding data distributions and yield breakthroughs in producing realistic results.

### Deep network architectures

The studies on the architectures of deep neural networks can be divided into two categories: improvements over RNN-based architectures and developments built upon attention mechanisms.

Gers *et al.* [86] proposed a variant of RNNs with forget gates to process long data sequences. In subsequent studies, this mechanism was further elaborated in the form of gated recurrent units [87], which included update gates and reset gates to control information flow. The update gate controls how much information is preserved, and the reset gate determines how much of the accumulated memory should be discarded. Networks that utilize the gated recurrent unit typically have fewer parameters and are computationally efficient; however, they are limited in handling long-term dependencies. Nonetheless, these characteristics make them suitable for real-time generative tasks.

Transformers [88] are the most influential deep architecture at present. They process input as tokens, which represent the basic units of data. In the case of textual data, tokens can be words, characters or bytes, depending on the tokenization method used. Using different tokenization methods [89–91], transformer-based models are capable of handling data from different modalities. Moreover, they can leverage large-scale training datasets, establishing themselves as state-of-the-art approaches for diverse applications, including generative tasks.

Transformers process sequential data through two key techniques: positional encoding and the self-attention mechanism. Positional encoding adds position information to the input embeddings, enabling the transformer to capture the relationships in sequential data. The self-attention mechanism assigns weights to different data elements and helps the transformer focus on the most useful parts of the data. This design allows transformers to capture long-range dependencies. Moreover, transformers are well suited for GPU-optimized operations, such as parallel computation, which leads to fast training and inference processes. Subsequent studies [90,92] effectively adapted transformers to vision tasks. Vision transformers divide images into fixed-size patches and utilize a linear mapping technique to convert the patches into a sequence, thus unifying the backbone architecture for visual and textual data. Other efforts have focused on improving the computational efficiency of transformers; examples include linearized self-attention [93,94], sparse transformers [95] and approximation approaches [96].

The attention mechanism has also inspired other network architectures in addition to transformers. For instance, the attention-based method introduced in [97] effectively models graph-structured data, including protein interactions. In addition, there are studies of deep architectures beyond the attention mechanism, such as capsule networks [98] and state space models [99].

### Deep generative models

Deep generative models refer to machine learning models based on deep networks. These models originate from various generative theories, and the most representative categories include generative adversarial networks, variational autoencoders and probabilistic diffusion models.

Generative adversarial networks (GANs) [100,101] have been widely applied to various generative tasks due to their capability to produce realistic data. GANs engage in a minimax game, where the generator aims to produce realistic samples, while the discriminator aims to differentiate between generated and real samples. According to game theory, both networks improve their performance through the adversarial training process, until they reach a dynamic equilibrium where the discriminator cannot distinguish between generated and real samples. This provides theoretical support for the superiority of GANs in terms of generation quality. GAN-based generative models [102–105] have further advantages, especially in terms of the controllability of generated content. For example, StyleGAN [104] can perform semantic editing on images at the pixel level. Additionally, the training and inference of GAN models are very fast, particularly in comparison to graphics-based methods. However, GANs suffer from model collapse, which means that the generator fails to fully capture the complexity of the data distribution, resulting in a restricted variety of generated data. Although some studies [106] have aimed to alleviate this problem, the training of GANs is still prone to collapse, particularly when the model is scaled up.

The variational autoencoder (VAE) [107] is another typical type of deep generative model. A VAE learns the distribution characteristics of high-dimensional data in a latent space. It utilizes an encoder network to map high-dimensional data to latent representations and a decoder network to reconstruct the data with resampled representations. During training, the VAE optimizes the reconstruction error while ensuring that the distribution of latent representations approaches a prior distribution. These approaches exhibit strengths that complement the capabilities of GANs. Theoretically, VAE-based models [108,109] can capture the entire distribution of data. Thus, sampling representations from the latent space offers diverse unseen data points. However, the generated data tend to be blurry, and thus lack realistic appearances.

Probabilistic diffusion models [37,38] describe data generation as a stochastic process. These models involve two processes: the forward diffusion process and the reverse process. During the forward process, prior noise is progressively added to the real data, and the model learns to predict the noise. Then, during the reverse process, the model transforms the sampled noise into data. Studies have focused on improving the speed of the reverse process, which includes introducing latent space generation [110], incorporating discriminative priors [111], combining model distillation techniques [112], etc. Diffusion-based approaches can utilize large-scale training data effectively and generalize well across various generative tasks. In particular, these methods have demonstrated unprecedented performance in zero-shot generative tasks, producing impressive scenes that do not exist in the real world. However, the training and inference of probabilistic diffusion models are computationally intensive. This results in computational demands that are orders of magnitude greater than those of GANs.

Deep generative models have gradually replaced traditional machine learning models since the late 2010s. The ability to utilize large-scale training data allows deep generative models significant flexibility in handling high-dimensional generative tasks. By that time, GAI-generated content had become realistic and was sometimes even indistinguishable from real content. Respective applications use transformer-based models to translate languages or generate various types of textual data, including documents, web pages and code. Moreover, deep generative models such as WaveNet [113] can synthesize realistic and comprehensible audio content, thus supporting multiple applications such as music generation and speech synthesis. FaceSwap [114], which manipulates media by replacing one person’s appearance with that of another, was created in 2017.

### Deep generative learning for graphics

The successful application of deep neural networks to 3D perception and understanding has inspired efforts to integrate these networks with rendering techniques. However, the traditional rendering process does not ensure differentiability with respect to model parameters. Therefore, some studies have developed differentiable rendering and end-to-end algorithms, which allow gradient-based parameter optimization and direct editing of 3D scenes.

The neural radiance field [7] is a typical differentiable rendering technique based on implicit representations. The cited study is based on the overall framework of ray tracing, uses a multilayer network to model the volumetric scene function and applies volume rendering algorithms to simulate the process of light travel. Moreover, Fridovich *et al.* [115] used sparse voxel representations to achieve computationally efficient rendering. The recently proposed 3D Gaussian splatting [116] utilizes a rasterization pipeline and employs neural point clouds as scene representations. Three-dimensional Gaussian splatting methods can meet the requirements of real-time rendering while generating realistic images.

Ray tracing algorithms have become popular in generative applications based on computer graphics due to improvements in computing equipment. In the 2010s, these algorithms brought realistic avatars to commercial films. The techniques of overlaying animated scenes with live-action footage were also developed, enabling high-fidelity interactive rendering. In addition, deep learning–based supersampling technologies enabled real-time ray tracing at the 4K resolution on a consumer GPU. The availability of these technologies led to the emergence of various virtual, mixed and augmented reality devices, depicting lifelike digital worlds.

## FOUNDATION MODELS

The term ‘foundational model’ was introduced in the report of Bommasani *et al.* [117]. It refers to a base model that is trained on broad data and can be adapted to a wide range of downstream tasks. Such a model is also known as a large X model, for example, a large language model.

Constructing foundation models aligns with the conceptual framework of classic model-based approaches, but does not mandate adherence to any particular type of generative model. The common approach today involves the use of deep neural networks, particularly transformers. As illustrated in Fig. 2c, the training schemes typically include generative pretraining and fine-tuning [8], with specific details varying depending on the input and output data modalities. Foundation models represent a significant shift in GAI, achieving extraordinary performance in the generation of texts, images and contents of other modalities.

### Large language models

Foundation models were first applied in the language domain. Devlin *et al.* [118] attempted to pretrain a model on large-scale unlabeled corpora and then fine-tune the network according to specific downstream tasks. Interestingly, researchers find that scaling pretrained language models often leads to emergent abilities on downstream tasks [119]. For example, a 175B-parameter model [9] can solve few-shot tasks through in-context learning, whereas a 1.5B-parameter model [120] cannot do so well. However, even fine-tuning such a large model is computationally expensive. Hence, Wei *et al.* [121] proposed instruction tuning to fine-tune foundation models on a collection of datasets described via instructions, substantially improving zero-shot performance on unseen tasks. In addition, some studies [122] have combined instruction tuning with human preferences and feedback. Notably, ChatGPT [1], which was developed based on the large language model of the GPT type [9], presents an amazing ability to converse with humans. Subsequently, interest in large language models has continued to surge, giving rise to numerous influential studies [123–126]. Among them, Gemini is a remarkable family of large models [123], which demonstrates state-of-the-art capabilities in reasoning and understanding across various benchmarks. The recently released DeepSeek models [127] have demonstrated remarkable capabilities, particularly in reasoning tasks, while significantly reducing computational costs.

Prompt engineering is an important technique for working with foundation models. It helps foundation models adapt to specific problems without a change of model parameters. There are typically two types of prompt engineering. One involves carefully designing good prompts for a specific problem. For example, context learning provides additional context, such as exemplars, to help models understand the problem. Kojima *et al.* [128] simply added ‘Let’s think step by step’ before each answer, which can achieve better performance. The other type of approach compels models to imitate the reasoning process of humans. For instance, the method in [129] provides a few chains of thought demonstrations as exemplars in prompting. The least-to-most method [130] breaks down a complex problem into a series of simpler subproblems and then solves them in sequence.

The success of large language models has advanced vision-language understanding, where the main point is aligning and fusing vision and language features. Researchers have proposed various methods with different architectural designs. The dual-encoder architecture [131] uses a parallel visual and language encoder with aligned representations. The encoder-decoder architecture [132] applies joint feature encoding and decoding sequentially. In addition, Alayrac *et al.* [133] used a large language model as an adapter, harnessing its superior capacity through visual prompts.

### Large text-to-image models

Numerous large text-to-image models have been built based on foundation models, achieving unprecedented breakthroughs. The text-to-image models are typically based on GANs [134], autoregressive models [135] or probabilistic diffusion models [136,137]. Their supervision for aligning the text-image features is obtained from large language models [138] or vision-language models [131]. Stable Diffusion [136] and FLUX [137] models are among the most commonly used text-to-image models and are known for their outstanding performance in generation and following textual instructions.

The text-to-image models facilitate various downstream tasks, including style transfer, personalization, semantic editing, image restoration, image enhancement, etc. These methods can be divided into three types: training-based, testing-time tuning and training-free approaches. The training-based approaches collect additional data and fine-tune the model. These methods include domain-specific editing with weak supervision [139], reference and attribute guidance via self-supervision [140] and instructional editing via full supervision [141]. Testing-time tuning methods optimize the model parameters during model inference; such methods include embedding optimization [142], hypernetwork guidance [143], latent variable optimization [144] and hybrid fine-tuning [145]. Training-free methods use off-the-shelf models without changing any model parameters. These approaches [146] refine the input texts or masks or alter the inverted latent code to generate outputs tailored to the specified task.

### Large text-to-video models

Some research efforts [110,147] have led to the development of large models for generating video content. These models are derived from text-to-image models, making them sub-derivatives of foundational models. Despite the variety of designs, most of the approaches follow the pipeline proposed in Sora [147]. This pipeline first generates low-dimensional videos or latent codes [110], which are then refined using temporal and spatial super-resolution techniques.

Current video generation models are extremely data intensive due to the staggering complexity of the state space in video data. As a result, effectively utilizing limited real-world data is crucial. Existing studies can be broadly categorized into two approaches: spatial-temporal compression [148] and efficient transfer learning [149].

Some studies [147,150] adhere to the scaling law and increase investments in data and computational resources, leading to superior video generation quality and early commercial successes. For example, the aforementioned Sora model [147] can simulate real-world object interactions and generate corresponding videos lasting several minutes. Video models [150] from Runway AI are capable of generating visual storytelling scenes. Produced without human intervention, these synthetic results achieve remarkably realistic effects.

### Expanding the use of foundation models

Foundation models have made prominent contributions to GAI. Moreover, applications based on such models can generate content beyond text and images. Because of their ability to generate textual instructions, large language models can help humans interact with computational software and even physical tools by natural language. This makes it possible for humans to use generative technologies without requiring specialized knowledge. For instance, Suno AI [151] allows users to generate realistic music through language descriptions, including customized voices and sound effects, while also designing album covers; First *et al.* [152] applied large language models to automated reasoning, using them to generate proofs. This effort revitalizes the domain of formal software verification and mathematical problem solving. Additionally, research communities have explored using large language models for specialized tasks, such as automated manufacturing [153], algorithm design [154] and molecular discovery [12].

Because of their generalized capabilities for generation, foundation models provide an intuitive way to simulate real-world scenarios. By learning patterns in complex environments, foundation models can predict environmental dynamics and generate decision-making strategies. In this context, they serve as world models [155] within a specific system. This facilitates the expansion of human comprehension, as the generated content can offer predictions about future events.

Foundation models have been applied to building world models in practical systems. For instance, studies on integrating foundation models into transportation systems [156] focus on addressing practical challenges, such as vehicle navigation and communication. By fulfilling personalized demand through automated content generation, these approaches enhance both the service quality and efficiency of transportation systems. Approaches that integrate foundation models into physical entities have also emerged [157,158]. These efforts leverage foundation models for action control. In addition, research on intelligent agents powered by foundation models is actively advancing [159]. Such generative technologies mitigate the need for highly detailed physical modeling, which is expensive in real-world scenarios.

### Limitations of foundation models

However, current GAI technologies are far from perfect. While foundation models are driving commercial success at an unprecedented pace, they still make mistakes in problems that are trivial for humans, much like the AI systems from 70 years ago did. For instance, as of this writing, an issue reported in the OpenAI community revealed that ChatGPT still incorrectly assumed that 9.11 was greater than 9.9 in a dialogue.

The example above illustrates the hallucination problem in foundation models, where they occasionally generate irrelevant, inconsistent or incorrect content. Such hallucinations can be highly misleading, causing users to believe that the provided information is accurate. In other instances, the generated content may be nonsensical, resulting in confusion among users. Additionally, foundation models are susceptible to reasoning errors, as demonstrated in the numeric comparison example, as well as factual inaccuracies, where some of the information is fabricated.

Diagnosing and fixing foundation models is challenging due to their black-box nature. The generative processes are not interpretable, as foundation models consist of complex neural networks trained on vast datasets. As a result, diagnosis is primarily based on outputs, without direct access to the decision-making processes. Such opacity leads to the current approach of applying case-by-case patches to address specific issues, while a general fix remains essentially difficult.

The bottleneck of computational resources presents another significant challenge. The hardware costs for developing foundation models are prohibitively high for individuals, academic institutions and even some AI research organizations. While some tiny versions of foundation models can be deployed on commercial-grade devices, the cost of training a foundation model with billions of parameters is measured in millions of dollars. Running foundation models on portable computing devices presents more challenges, particularly in resource management, computation offloading and mobility management, among others [160]. OpenAI reports that updating a large model can take several months due to limited computational power, meaning that such models cannot incorporate real-time information or receive timely updates.

Harnessing the capabilities of foundation models, which aims to avoid generating illegal, immoral, biased or inaccurate information, is becoming increasingly challenging. Several serious issues have been identified in applications built on these models. For instance, AI chatbots can be tricked by carefully crafted prompts into leaking sensitive data, such as individuals’ names, phone numbers and addresses. Additionally, the content generated by large models can be misused, but preventing such misuse is difficult due to gaps in current security policies.

## FUTURE DIRECTIONS

GAI applications based on foundation models have become the current mainstream practice, but the vulnerabilities of foundation models have also been inherited. Currently, research on GAI safety lags behind its technological development. We summarize several critical security issues that urgently require further attention and development.

*Value alignment.* GAI should understand human intentions and adhere to human values, ensuring that the generated content is helpful while preventing misuse for inappropriate purposes. This goal promotes the practices of responsible GAI and requires a deeper understanding of evaluating alignment capabilities [161]. It also necessitates the development of more comprehensive guidelines that accurately reflect human preferences, which presents a significant challenge to current statistics-based evaluation paradigms.

*Source identification.* Current GAI-generated content is convincing and easy to manipulate. This makes it necessary to ensure that the origin of generated content is traceable to prevent intellectual property disputes. Therefore, techniques such as invisible watermarks and signatures should be further studied to verify the integrity and ownership of the generated content. However, since GAI-generated content is highly malleable, imposing unalterable identifiers without negatively affecting usability presents a significant challenge.

*Security regulations.* GAI developers should adhere to a necessary consensus, ensuring that their products do not harm humanity. On the one hand, standards organizations should require that the development process follows legal and ethical guidelines, similarly to the review process in scientific research. On the other hand, a correction mechanism must be established to prevent the dissemination of harmful GAI models or generated content. Depending on the circumstances, this mechanism should mandate that developers publicly verify, correct or retract any released GAI technologies that fail to meet these standards.

Despite safety concerns, the current wave of interest in GAI is likely to persist. Here, we discuss several promising directions with the potential to result in breakthrough improvements and address a broader range of human needs. We note that other dimensions, which may not yet have received broad attention, are also worthy of further exploration.

*Unification of modalities.* Although there have been some achievements in bridging the textual and visual domains, research on the interactions among multiple systems [162] is still in its early stages. How to align and fuse multiple modalities, including text, images, videos and structured data from different systems, remains an open challenge.

*Deciphering GAI models.* Explaining how GAI models work, particularly the decision-making process, in a way that is understandable to humans, is both challenging and indispensable. Current efforts [163] have provided heuristic understanding based on phenomenological approaches. Moreover, introducing interpretable principles based on theories from domains such as thermodynamics [38] and electrodynamics [164], into AI modeling offers promising directions for enhancing the transparency of GAI, with initial successes already demonstrated. However, the reliability of this understanding should be grounded in a computational logic framework to ensure more accurate and dependable interpretations.

*Learning from GAI-generated content.* Synthetic data are becoming increasingly accessible and important. While current studies assume that synthetic data primarily represent interpolations of patterns from existing data, GAI models have demonstrated the ability to make nontrivial inferences for specialized tasks. Moreover, GAI models can reduce labeling costs, augment existing datasets and facilitate the learning and understanding process of humans. Consequently, more extensive research is anticipated to explore the use of synthetic data, potentially making such data a dominant resource in the future.

*Supervision beyond human capability.* Throughout the history of GAI, researchers have utilized knowledge familiar to humans to develop generative models. A recently released model [165] trained through reinforcement learning without supervised data has demonstrated reasoning capabilities on par with top-performing models that rely on human supervision. This finding suggests that foundation models may develop superhuman capabilities through self-enhancement. Assuming that these models eventually achieve such capabilities, how can humans effectively regulate them? It is thus necessary to change the learning paradigm while adhering to the principles of serving humanity.

## CONCLUSION

This work summarizes the historical and ongoing developments of GAI. We divide the methodologies into rule-based generative systems, model-based generative algorithms, deep generative methodologies and foundation models, and introduce their characteristics and applications. The focus is not on reviewing all the relevant literature, but rather on providing a brief summary of representative methodologies, emphasizing general principles and strategies rather than specific algorithms. Many strategies and ideas mentioned in this work can be realized in various forms, possibly with additional advantages in the future. We also discuss the remaining issues in the context of existing approaches. Moreover, we introduce some potential research directions to address the risks that may undermine the development of GAI.
